# Palmar plating for displaced metacarpal neck fractures: A new technique

**DOI:** 10.1051/sicotj/2026037

**Published:** 2026-10-15

**Authors:** Ahmed Naeem Atiyya, Moheeb Gadullah, Hamdy E. Alnaggar, Khaled Nabil Youssef, Ahmed Gamal Nouh, Ahmed Morsi

**Affiliations:** 1 Department of Orthopaedic Surgery, Ain Shams University Hospitals Cairo Egypt; 2 Department of Orthopaedic Surgery, Helwan University Hospitals Cairo Egypt

**Keywords:** _Metacarpal fractures_, _Palmar plating_, _Hand surgery_, _Fixation techniques_, _Functional outcome_

## Abstract

*Purpose:* To evaluate fixation of metacarpal neck fractures using miniplates applied via a palmar approach as an alternative to conventional dorsal plating, which carries a higher complication rate. *Methods:* A prospective case series of 34 patients with acute unstable metacarpal neck fractures treated with palmar miniplating from 2018 to 2022. *Results:* The mean follow-up was 25.79 months (range, 22–36 months). All fractures united in a mean of 5.4 weeks. Grip strength averaged 96% ± 4.01%, and ROM averaged 93% ± 4.25% compared to the contralateral hand. Minor complications occurred in 11.76% of cases. *Conclusion*: In the short term, palmar miniplates for unstable metacarpal neck fractures result in excellent radiological and functional outcomes with minimal complications.

## Introduction

Metacarpal fractures represent approximately 40% of all hand fractures [1]. Among these, the fifth metacarpal is most commonly affected, accounting for nearly 20% of all hand fractures [2], followed by the fourth, third, and second metacarpals. Metacarpal neck fractures are particularly prevalent among males, especially athletes and manual laborers [3]. These injuries typically result from a direct blow to a clenched fist, such as in boxing, leading to a characteristic fracture pattern: dorsal apex angulation with palmar comminution at the neck. The flexed fracture position is attributed to deforming forces exerted by the interosseous muscles, which originate from the metacarpal shafts and insert into the proximal phalanges [4]. There is currently no established consensus on the optimal surgical management of metacarpal neck fractures. Several fixation techniques have been described, each with specific advantages and limitations. Dorsal plating, for example, provides stable fixation that permits early mobilization [5], Dorsal plating has been associated with a relatively higher complication profile, including extensor tendon irritation, adhesions, hardware prominence, joint stiffness, and the need for secondary procedures such as hardware removal or tenolysis. Reported complication rates vary across the literature, reaching 30–36% in earlier studies [6, 7]. However, more recent studies using low-profile miniplates suggest improved soft tissue tolerance and potentially lower complication rates. Therefore, concerns regarding tendon-related complications remain relevant, particularly in conventional dorsal approaches. Given that most metacarpal neck fractures exhibit palmar displacement with dorsal apex angulation, and considering the palmar concavity of the metacarpal shaft and neck – which positions the flexor tendons relatively away from the bony surface – we hypothesized that applying low-profile mini plates from the palmar side could provide adequate fixation while minimizing soft tissue irritation. We further speculated that this approach would lower plate removal to a minimum.

## Methods

### Study design and patients

A prospective case series was conducted on 34 patients who underwent surgery for metacarpal neck fractures between June 2018 and August 2022. The study protocol was approved by the institutional review board, and written informed consent was obtained from all participants.

### Inclusion criteria

Patients with acute, unstable, and displaced fractures of the metacarpal neck were included. Acute fractures were defined as injuries treated within 10 days of the time of trauma. Fractures were considered unstable if clinical or radiographic signs of instability were evident within 10 days of injury. Clinical signs included either rotational deformity (leading to visible finger scissoring) or pseudo-clawing (defined as a proximal interphalangeal joint [PIPJ] extension lag >30°). Radiological criteria for instability included apex dorsal angulation exceeding: (i) 15° in the second and third metacarpals, (ii) 30° in the fourth metacarpal, (iii) 45° in the fifth metacarpal. Fracture angulation was measured using the sub-capital axis angle method in the oblique hand view, as described by Braakman [8]. This angle is defined between the sub-capital and shaft axes: the sub-capital axis passes through the midline of the neck using the “true-eye” technique, and the shaft axis is drawn from points at one-third and two-thirds of the metacarpal length.

### Exclusion criteria

Patients with open dorsal wounds, active infections, or skeletal immaturity were excluded. Patients with previous fractures or prior surgical intervention involving the same metacarpal were also excluded. Demographic data for all patients are summarized in Table 1.

**Table 1:** Radiological outcomes.

Parameter	Result
Average angulation correction	Consistent with Ali et al. [9]
Radiological union rate	Comparable to Foucher [10]
Residual deformity	Minimal across cases

### Surgical technique

All surgeries were performed under general anesthesia and pneumatic tourniquet, except in three patients who received WALANT (Wide Awake Local Anesthesia No Tourniquet) anesthesia. Skin incisions were tailored to the affected metacarpal. In radial metacarpals, incisions were aligned with palmar creases; in ulnar metacarpals, zigzag incisions were more common (see Figure 1). Meticulous dissection was performed to identify and protect the neurovascular bundles and flexor tendons. Partial release of the interossei muscles was done to expose the fracture site. Fracture reduction and fixation were performed using titanium low-profile mini plates (1.5 or 2.0 mm), including non-locking or combi-hole systems depending on availability. Plates were pre-contoured in all cases, with increased bending for ulnar metacarpals. The required bending angle was determined using the contralateral metacarpal’s sub-capital axis angle as reference. In many cases, the pre-bent plate aided fracture reduction indirectly. The plate was positioned in the flare of the metacarpal neck, strictly proximal to the “limit line” – a line passing through the widest part of the metacarpal head in the lateral view, corresponding to the end of the cartilage cap as per Braakman [8]. Over-distal placement was avoided, particularly in distal fractures, to prevent impingement with the proximal phalanx during metacarpophalangeal (MCP) joint flexion. Two to three screws were inserted proximally. Passive finger flexion was used intraoperatively to verify the absence of impingement. Fluoroscopic imaging was used to confirm fracture reduction, plate position, and screw length (Figures 2–4). Skin closure was performed using non-absorbable 4–0 sutures.

**Figure 1:**
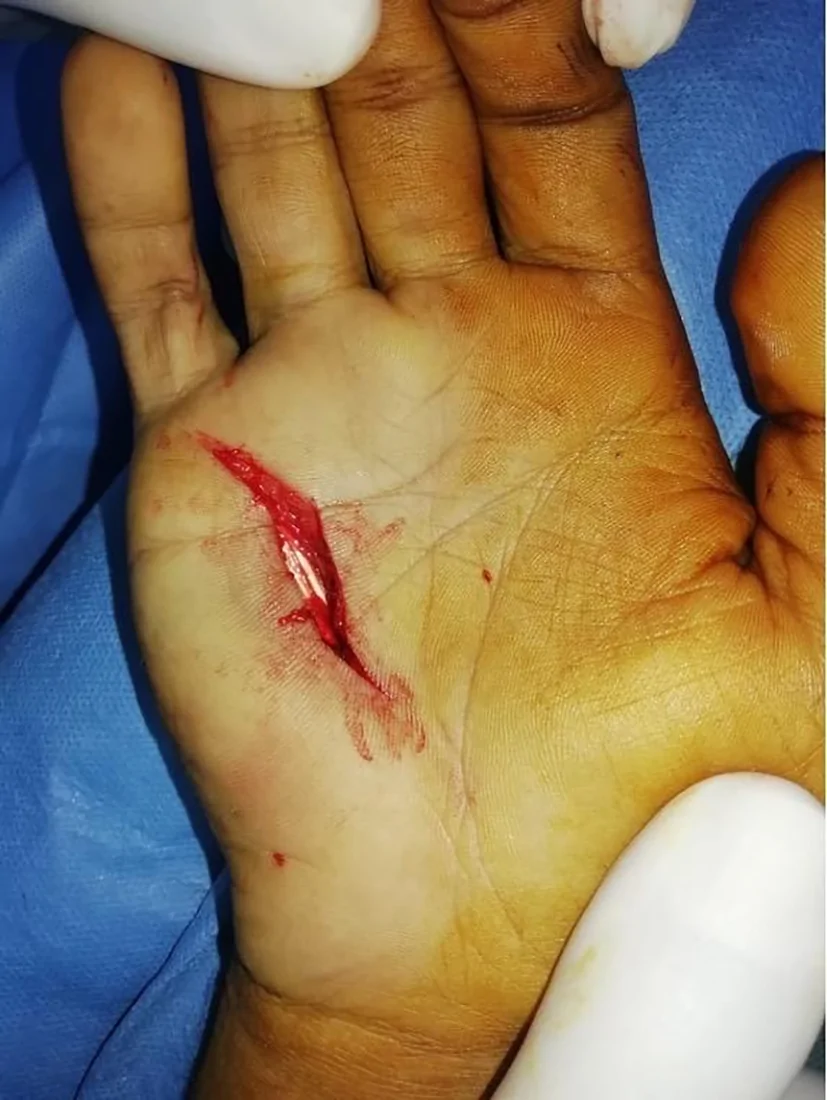
Figure shows the skin incision over the 5th metacarpal.

**Figure 2:**
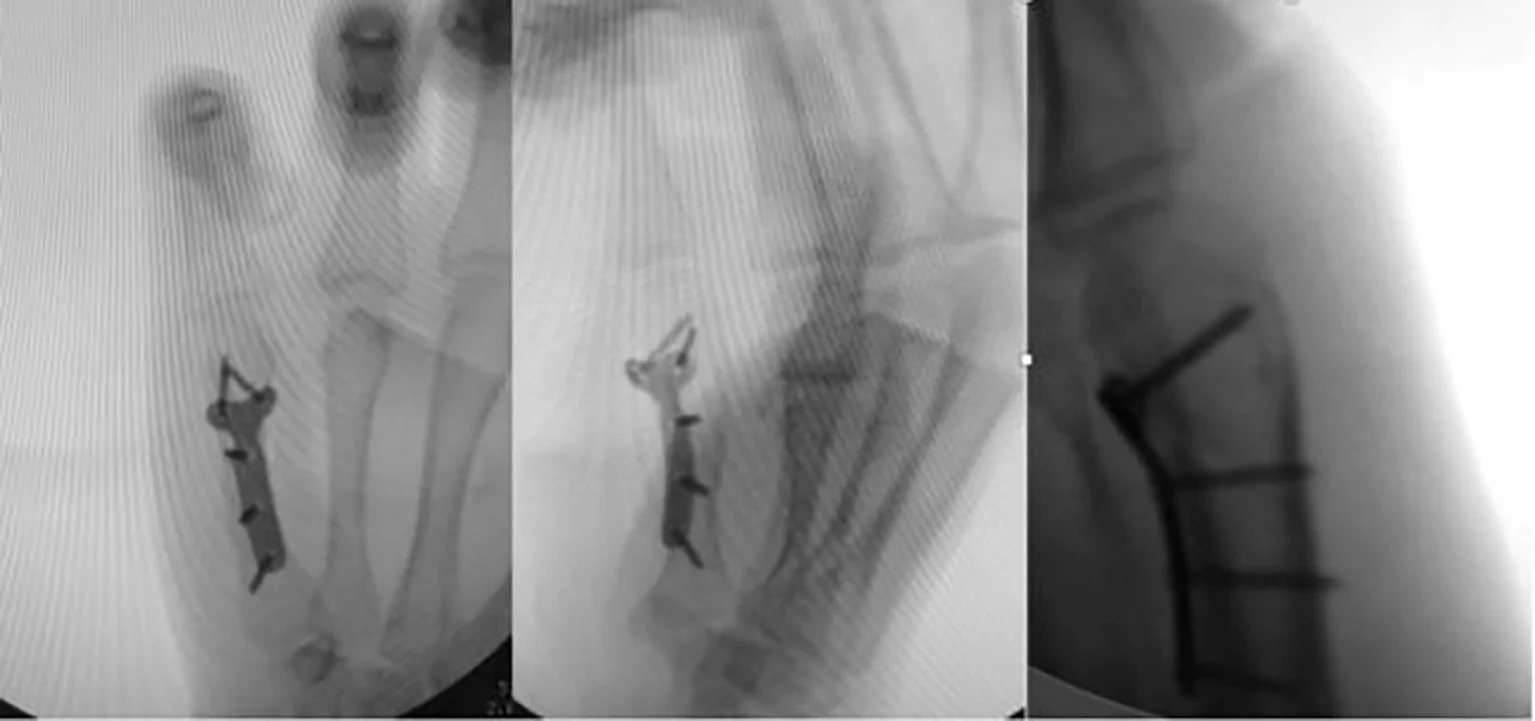
Intraoperative imaging of a 5th metacarpal neck fracture (AP and oblique views).

**Figure 3:**
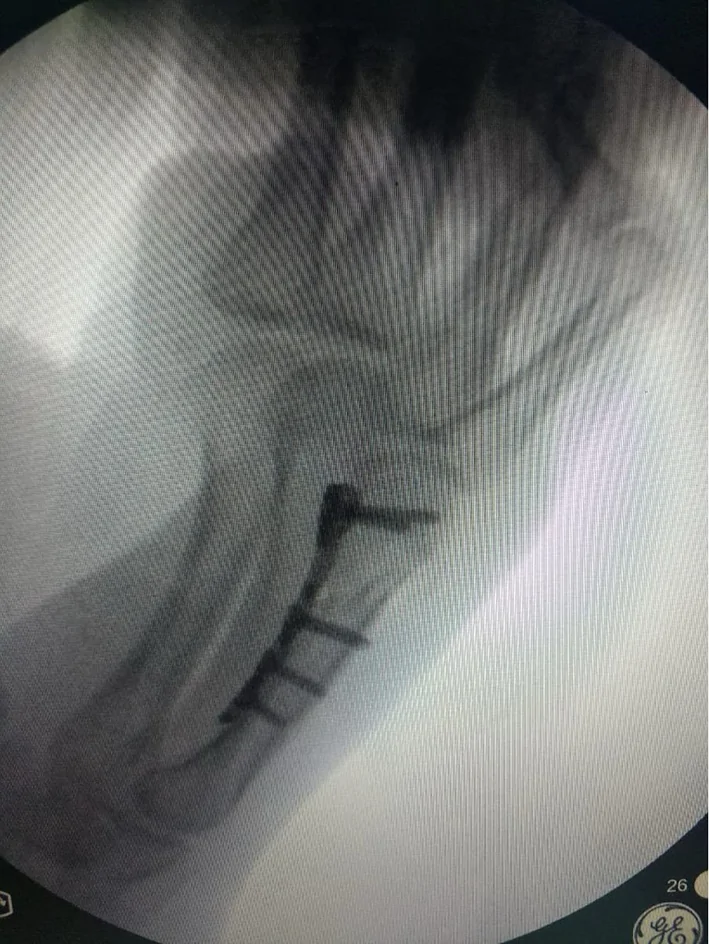
Lateral intraoperative imaging of a 5th metacarpal fracture fixed with a palmar plate.

**Figure 4:**
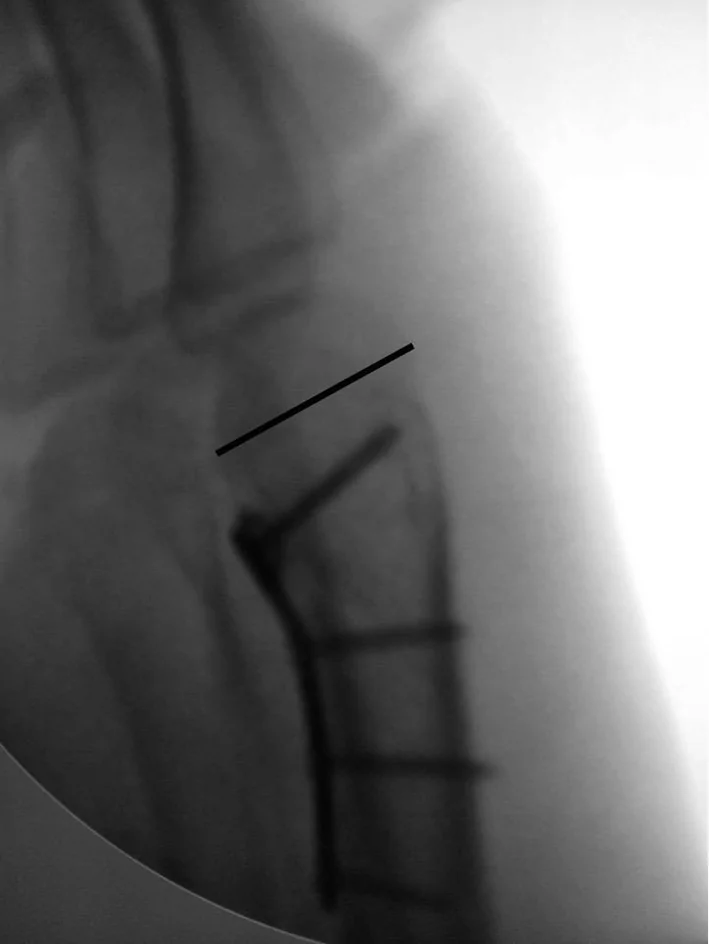
Shows lateral view intraoperative image of the metacarpal and the distal limit line of the plate (black line).

### Postoperative rehabilitation

A palmar below-elbow slab extending to the proximal phalanx was applied for the first five postoperative days. Patients were encouraged to perform active range-of-motion (ROM) exercises of the PIPJ, distal interphalangeal joint (DIPJ), elbow, and shoulder from day one. On day five, the slab was replaced with a removable splint, and buddy taping to the adjacent finger was initiated. Full active finger and wrist ROM was encouraged on an hourly basis. By the end of week three, the splint was discontinued, and passive ROM exercises were added. Progression to resisted exercises was primarily guided by clinical assessment, including pain resolution and functional recovery, rather than relying solely on radiographic evidence of union.

### Evaluation

Patients were evaluated both clinically and radiologically. (i) Pain was assessed using the Numerical Pain Rating Scale (NPRS, 0–10) [11]. (ii) Total active motion (TAM) was measured using a goniometer and expressed as a percentage of the contralateral hand. (iii) Grip strength was evaluated using a Jamar hydraulic dynamometer, also expressed as a percentage of the contralateral side. Radiographic assessment included fracture union and residual angulation, calculated using the sub-capital axis angle on oblique hand views as described by Braakman [8]. Postoperative radiographs (PA and oblique views) were obtained immediately, then weekly starting from week 3 until union was confirmed (Figure 5). Figure 6 shows clinical ROM 12 months postoperatively in a patient with a second metacarpal fracture.

**Figure 5:**
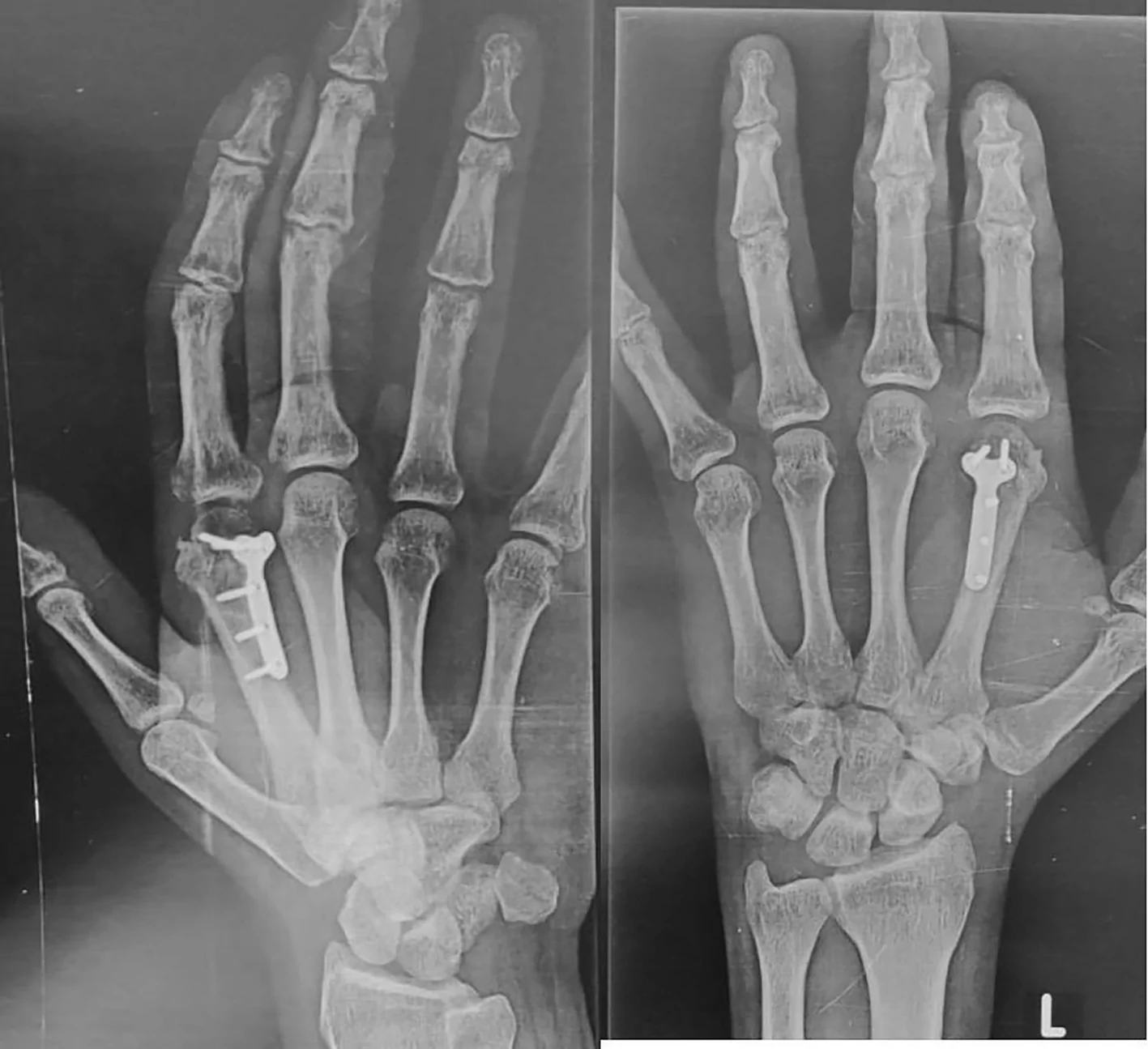
Showing healing of a case of 2nd metacarpal neck fracture at 6 weeks follow-up.

**Figure 6:**
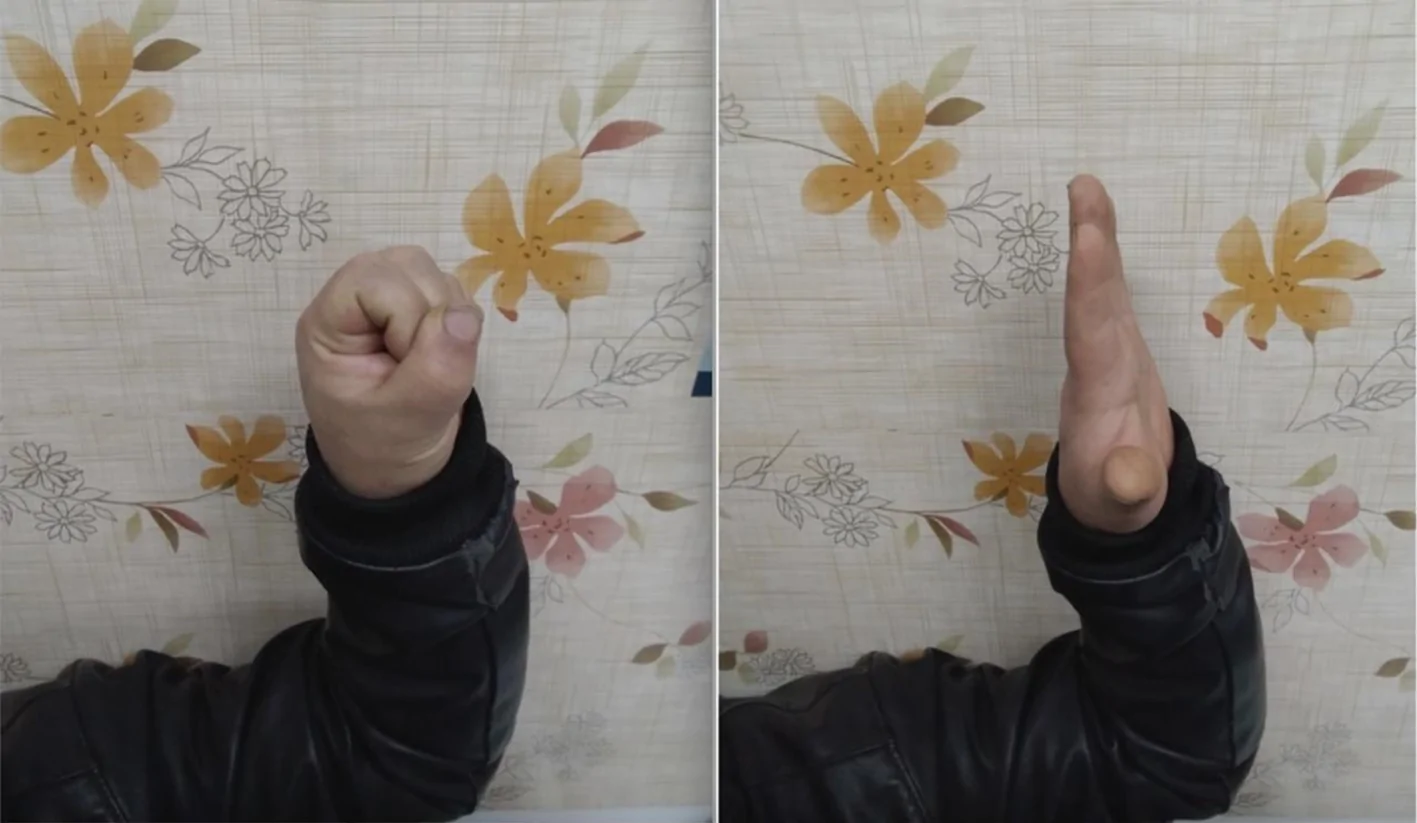
Clinical picture showing the ROM of a patient operated 2nd metacarpal at 12 months follow-up.

### Statistical analysis

Descriptive statistics (mean, standard deviation) were used for follow-up duration, union time, NPRS, TAM, grip strength, and fracture angulation. Pre- and postoperative angulation values were compared using paired *t*-tests. A *p*-value <0.05 was considered statistically significant.

All methods were carried out in accordance with relevant guidelines and regulations.

## Results

A total of 34 metacarpal neck fractures were operated on between 2018 and 2022. The cohort included 29 male and 5 female patients, with a mean age of 33.73 years (range, 22–50 years). The average interval from injury to surgery was 2.91 days (range, 1–10 days), and the mean follow-up duration was 25.79 months (range, 22–36 months). The mean operative time was 47.7 min (range, 35–60 min). All fractures achieved union, with a mean healing time of 5.4 weeks (range, 4–8 weeks). At final follow-up, grip strength averaged 96% ± 4.01% compared to the contralateral hand, while the total active range of motion (ROM) reached a mean of 93% ± 4.25% relative to the uninjured finger.

Radiographically, the mean preoperative sub-capital shaft angle was 64.26° (range, 30°–80°), which improved significantly postoperatively to a mean of 9.70° (range, 5°–20°) (*p* < 0.05).

Pain scores, assessed using the Numerical Pain Rating Scale (NPRS), averaged 0.7 (range, 0–2) at final follow-up.

There were no major complications requiring surgical revision. Minor complications occurred in 11.76% of cases (4/34), including one case of 5° malrotation, which was clinically accepted by the patient, one case of superficial wound infection successfully managed with oral antibiotics, and two cases of transient paresthesia that resolved spontaneously. No cases of hardware prominence, tendon irritation, or need for implant removal were observed. A summary of the clinical and radiological outcomes of the present study is presented in Table 2.

**Table 2:** Clinical and radiological outcomes of the present study parameter.

Parameter	Value
Union rate	100%
Mean time to union	5.4 weeks
Grip strength	96% ± 4.01%
Range of motion (TAM)	93% ± 4.25%
Complication rate	11.76%

## Discussion

Dorsally applied mini plates are usually used for osteosynthesis of comminuted, displaced metacarpal neck fractures. The approach is mostly used as it is simple and relatively safe. It needs minimal dissection with no critical neurovascular structures on the way to the bones [12]. Nevertheless, the complication rate with dorsal plating is not inconsiderable. It may reach up to 36%, usually related to extensor tendon irritation, limiting their excursion and necessitating secondary operations on many occasions.

Applying plates on the lateral surface might not be a suitable option for all the metacarpals. Moreover, it showed low mechanical strength even when compared with K-wires, regardless of the type of plate used [13].

The longitudinal arch of the hand is partly the gift of the palmar concavity of the metacarpals. This creates a hollow cavity in the palm in which the flexor tendons and the neurovascular bundles are superficially located away from the metacarpal bone. On the contrary, the extensor tendons lie very near to the dorsum of the metacarpal shafts.

The dorsal plates are applied on the tension side of the metacarpals. Nevertheless, the palmar comminution common with these fractures may imply that there may be no sufficient bony buttress in the opposite cortex to the plate to prevent cyclic bending, which is a prerequisite for the tension band principle [14].

Intramedullary fixation techniques, particularly headless compression screws, have gained popularity due to their minimally invasive nature and low hardware irritation rates. However, they may be less suitable in comminuted or unstable fracture patterns where plate fixation provides better control of alignment and rotational stability.

Considering the above anatomical and pathomechanical factors, a new idea of fixing unstable neck fractures of the metacarpals through a palmar approach instead of the commonly used dorsal approach was introduced. The previously mentioned advantages of the palmar plates may make them better for the buttressing effect for these palmarly angulated fractures. This advantage may allow for an earlier rehabilitation program. Moreover, these plates will be seated in the concavity of the metacarpal away from flexor tendons and neurovascular structures, thus reducing the risk of hardware irritation.

Nazerani et al. introduced the palmar approach for fixation of metacarpal shaft fractures in 2022. They reported their results of internal fixation of 14 metacarpal fractures either by screws or mini plates. Time to union and union rate were not clearly mentioned in their report. Nevertheless, all patients regained full ROM by 3 months and full power grip by 6 months, which was the final follow-up period. In this study, we utilized the palmar approach exclusively for metacarpal neck fractures. We did not report any loss of ROM or grip strength by the final follow-up [15].

The results of dorsal plating in the literature show a mean grip strength of around 86%, an active ROM of MPJ of around 72.2° ± 9.6°, and a postoperative angulation with a mean of 23.6° ± 9.7°. We obtained a comparable grip strength rate and a better correction of the fracture as reflected by the better correction of the apex dorsal angulation. Moreover, the palmar plate showed a lower complication rate of 11.76%, all of which were minor, compared to higher major complication rates in dorsal plates (up to 36%) [16–18].

Throughout the whole follow-up period, no patients needed any secondary operation for plate removal or tenolysis. This may be the main advantage of the palmar plates in comparison to the dorsal. It is speculated that the higher incidence of hardware-related complications, especially adhesions in dorsal plates, may imply that the range of motion is primarily lower than that of palmar plates.

Tsung-Yu et al. conducted a biomechanical study comparing the rigidity of both the palmar and dorsal locked plates used to fix metacarpal neck fractures with a simple transverse fracture in an artificial model. According to them, the yield strength and stiffness of the dorsal plates were higher than those of the volar ones. Nevertheless, the activity of daily living is usually less than the yield strength reported in their study. They suggested less aggressive rehabilitation in the early postoperative weeks for the volar plates to avoid hardware failure. In our study, early motion was started after the soft tissue swelling subsided. We did not report any plate or screw failure [19].

We acknowledge that there is a risk of injuring neurovascular bundles during the approach. Meticulous dissection is of paramount importance, which resulted in a low rate of minor complications. Only two patients in this study had transient paresthesia, which resolved spontaneously in 2 and 4 weeks. The technique requires a learning curve for less experienced surgeons to be conducted safely. However, we believe that it is not a steep curve. Meanwhile, the surgical approach is relatively familiar to hand surgeons and does not need any extensive training.

We elected for the distal margin of the plate not to exceed the line through the widest part of the head of the metacarpal (the limit line). The plate and the distal screws can buttress the fracture without impingement on either the proximal phalanx or the flexor tendons. This situation is similar to the Soong classification in distal radius fractures fixed with palmar plates [20].

Screw protrusion through the dorsal cortex can cause tethering of extensor tendons. Feeling any protruding tips of the screws on the dorsal cortex and checking the appropriate length by intraoperative fluoroscopy are essential steps to guard against this risk. The second tip is to direct the drill bit either radially or ulnarly to the extensor tendons during drilling.

The scars were inconspicuous in most cases. The scars in the radial digits were the best in this context. The incision was easily planned to overlie the thenar and palmar creases. The incisions were sufficient and left no visible scars. On the ulnar side, we tried to follow the proximal and distal palmar creases in addition to the adjacent, incomplete, thin creases as much as we could. The scars were minimal, and the patients were satisfied with them.

To the best of our knowledge, this study may be the first to use volar plating in fixing neck metacarpal fractures. The study is a prospective case series with a reasonable follow-up period. The number of patients recruited is one of the largest in the literature.

However, some methodological limitations exist. The level of evidence is relatively low due to the absence of a comparative group (e.g., dorsal plate fixation), which reduces the ability to definitively conclude superiority. Although we attempted indirect comparisons with published literature, future prospective comparative or randomized studies are warranted. A comparative table summarizing outcomes from published dorsal plating studies versus our results would strengthen external validity.

Additionally, no power analysis was conducted prior to patient enrollment. Although such analysis is not mandatory in prospective case series, acknowledging this limitation reflects methodological awareness. Blinding was not possible for the surgeon performing the procedures, and the outcome evaluation was done by the same investigator. While this may introduce observer bias, adherence to a predefined assessment protocol helped mitigate this risk. Future studies may benefit from independent outcome assessors and multicenter enrollment for broader generalizability.

## Conclusion

Palmar plating for unstable metacarpal neck fractures offers a safe, effective alternative to dorsal plating, achieving excellent functional outcomes and reducing the need for reoperation.

## Data Availability

Available from the corresponding author upon reasonable request.
